# Accomplices of the Hypoxic Tumor Microenvironment Compromising Antitumor Immunity: Adenosine, Lactate, Acidosis, Vascular Endothelial Growth Factor, Potassium Ions, and Phosphatidylserine

**DOI:** 10.3389/fimmu.2017.01887

**Published:** 2017-12-21

**Authors:** Peter Vaupel, Gabriele Multhoff

**Affiliations:** ^1^Department of Radiation Oncology, Klinikum rechts der Isar der Technischen Universität München, Munich, Germany

**Keywords:** antitumor immunity, adenosine, acidosis, lactate, vascular endothelial growth factor, phosphatidylserine, tumor hypoxia, potassium ions

## Abstract

In this minireview, we aim to highlight key factors of the tumor microenvironment, including adenosine, lactate, acidosis, vascular endothelial growth factor, phosphatidylserine, high extracellular K^+^ levels, and tumor hypoxia with respect to antitumor immune functions. Most solid tumors have an immature chaotic microvasculature that results in tumor hypoxia. Hypoxia is a key determinant of tumor aggressiveness and therapy resistance and hypoxia-related gene products can thwart antitumor immune responses.

## Introduction

Tumor angiogenesis, a key “hallmark of cancer” (1), is necessary for a sufficient supply of solid tumors with nutrients and oxygen and removal of metabolic waste products during tumor progression (2). The tumor as well as its *“immunological” microenvironment* including specialized lymphocytes and myeloid cells which are attracted by tumor cells contribute to vessel growth primarily *via* vascular endothelial growth factor (VEGF), but also exert immunosuppressive activities. Due to rapid tumor growth and an overload of proangiogenic factors, tumor vessels are often immature with poorly interconnected endothelial cells, leaky membranes, dead ends, and loosely attached pericytes (3). This chaotic tumor microvasculature can result in an inefficient blood-borne delivery, uneven distribution, and compromised penetration and distribution of macromolecules (e.g., antibodies, cytokines) and immune cells from tumor microvessels through the interstitial space to cancer cells. To reach all viable tumor cells in an effective dose, macromolecules and antitumor immune cells are negatively affected by several barriers to vascular, transvascular, and interstitial transport. A detailed description of these barriers has been previously presented (4–8).

*Vascular transport* includes the convective transport within abnormal vascular networks, significant arteriovenous shunt perfusion, and pronounced spatio-temporal heterogeneities. *Transvascular transport* of macromolecules is hindered by an impaired transluminal convective transport (extravasation) caused by an elevated interstitial fluid pressure (IFP 5 to 40 mmHg in tumors vs. −3 to +1 mmHg in most normal tissues) and intravasation back to the vascular compartment due to critically high IFPs, i.e., “back convection” from the interstitial space into the circulation. Interstitial hypertension additionally hinders *interstitial transport* of antibodies, delivery of cytokines and immune cells with antitumor activity through stopping, and even reverting of the microvascular blood flow, diversion of blood flow from the center to the periphery of tumors. Enlarged interstitial volumes, increased interstitial transport distances, and a reduction of the hydrostatic pressure gradient between intravascular space and interstitial compartment further impair an adequate delivery [for a review see Ref. (8)].

Newly formed microvessels in most solid tumors do not conform to the morphology of the vasculature of normal tissues. Tumor microvessels show many structural and functional abnormalities (5). These abnormalities not only directly or indirectly cause the abovementioned “biophysical” barriers for delivery of antitumor immune therapies but also have a negative impact on oxygen delivery to solid tumors (with substantial spatial and temporal heterogeneities). As a consequence, the metabolic *tumor microenvironment* (TME) is characterized by a critical oxygen (O_2_) depletion (hypoxia, anoxia), extracellular acidosis, substantially elevated adenosine (ADO) and lactate concentrations, and nutrient deprivation (4–8).

Hypoxia crucially contributes to genetic instability, intratumoral heterogeneity, malignant progression, tumor stem cell maintenance, sustained angiogenesis, development of treatment resistance, and metabolic reprogramming upon triggering the switch to HIF-1α-dependent phenotypes (9–11). In addition, tumor hypoxia/hypoxic stress and downstream effects of HIF-1α-activation can serve as major drivers for recruitment, activation, polarization, and expansion of immune-suppressive stromal cell populations causing an impediment to antitumor (innate and adaptive) immunity and cancer immunotherapy. In this minireview, the role of major “HIF-downstream factors” of the TME and egress of intracellular K^+^ upon tumor cell death inhibiting local functions and survival of immune cells, thus leading to tumor immune escape, will be discussed.

## Hypoxic Stress Factors Counteracting Local Antitumor Immune Responses

Earlier *in vivo* investigations of the effects of components of the TME on gene expression have elicited a substantial downregulation of a large number of microRNAs that are associated with the regulation and function of the immune system in hypoxic tumor areas (e.g., accumulation of cytotoxic CD8^+^ T cells in highly vascularized, normoxic areas vs. exclusion of cytotoxic CD8^+^ T cells from viable hypoxic tumor areas *in vivo*) raising the possibility that hypoxic tumor regions represent an immune-privileged tumor niche (12).

Major factors involved in local immune-suppressive actions exerted by hypoxia-/HIF-driven downstream factors include (a) ADO generation and accumulation in the extracellular space, (b) lactate accumulation, (c) extracellular acidosis, (d) overexpression of VEGF and activation of VEGF receptor (VEGF-R), and (e) externalization of phosphatidylserine (PS) on the outer membrane leaflet (13).

## ADO Inhibits Antitumor Immune Responses

**Figure 1:**
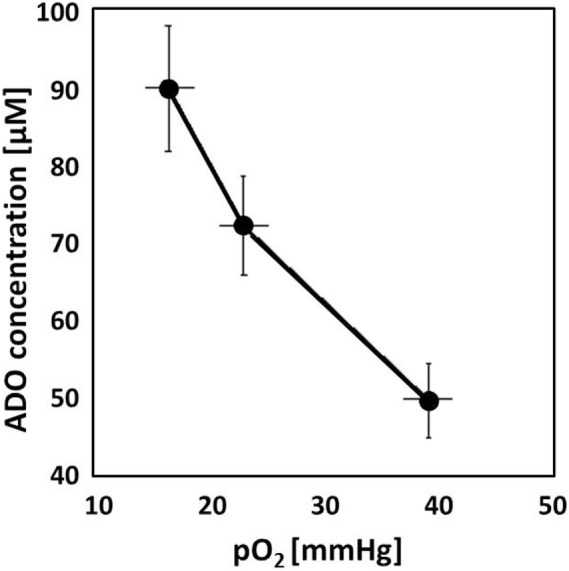
Adenosine (ADO) concentration measured in experimental tumors (DS-sarcomas, *n* = 26) as a function of the tissue oxygenation status. With decreasing mean tumor pO_2_ values, ADO accumulates in the tumor reaching ~100 µM in severely hypoxic tumors. For comparison, ADO levels in normal tissues are in the range of 10–100 nM (16, 17).

**Figure 2:**
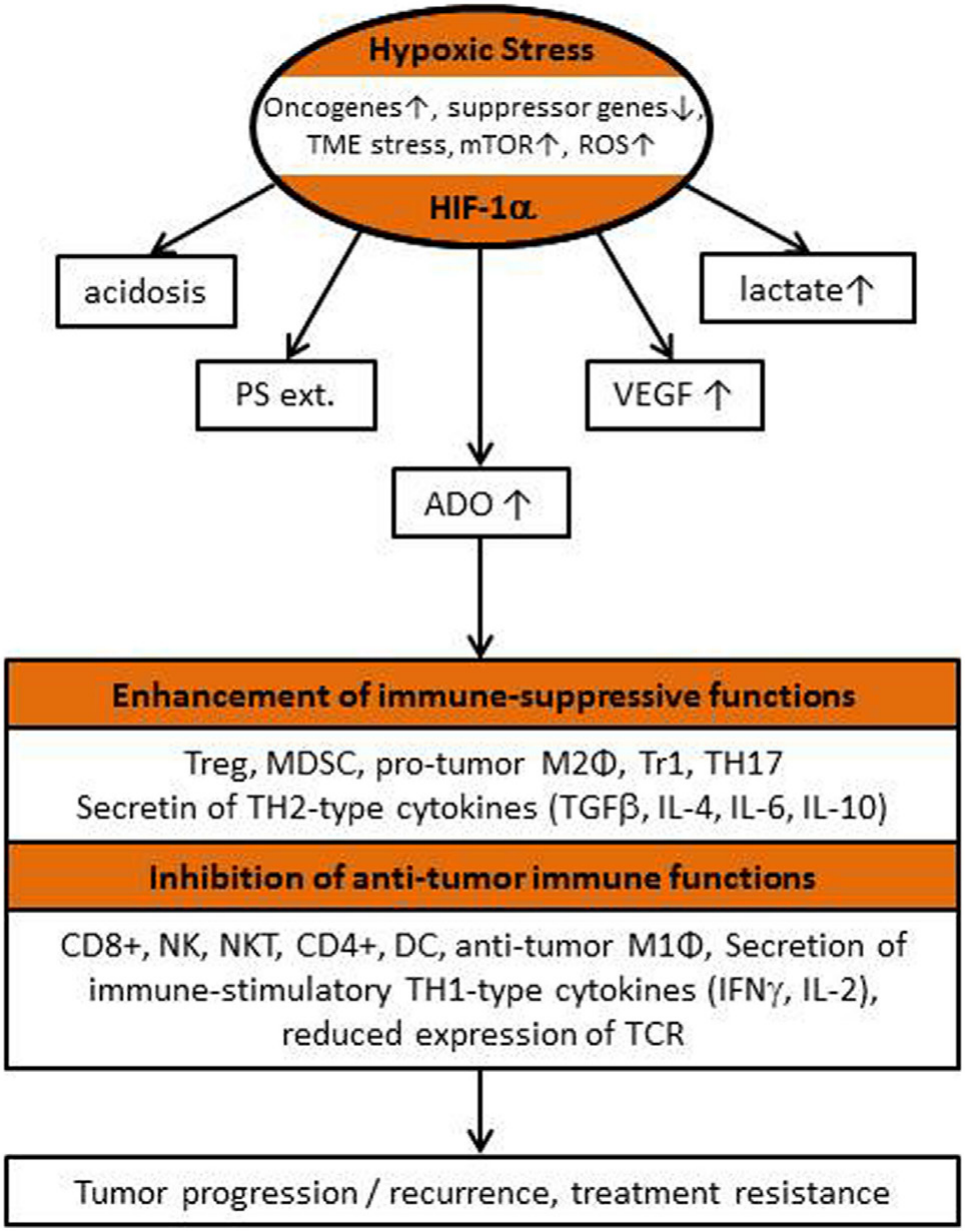
Flow chart describing “classical” hypoxia-/HIF-1α-driven features of the tumor microenvironment (TME) responsible for the local inhibition of antitumor immunity, for tumor progression/recurrence and poor patient outcome (see also list of abbreviations).

Adenosinergic effects on cancer, stromal, and immune cells have been summarized recently (14, 15). Briefly, upon hypoxic stress, cancer cells release ATP^4−^ through PANX-1 channels and/or exocytosis followed by ADO generation with subsequent ADO accumulation in the (positively charged) extracellular space of hypoxic tumor cells (10–100 µM, extracellular ADO). For comparison, normal tissues exhibit extracellular ADO levels in the range of 10–100 nM. ADO generation is mainly controlled by the activity of HIF-sensitive, membrane associated “tandem ecto-enzymes” CD39/CD73. Accumulation is further supported by an inhibition of the “downhill” ADO-re-uptake transporter ENT-1, and by an inactivation of the ADO-to-inosine conversion (through inhibition of ADO-deaminase). In severely hypoxic tumors regions, ADO concentrations up to 100 µM have been measured (Figure 1). Inhibitory effects of free ADO on innate and adaptive immune responses are multifactorial with a major emphasis on the proliferative and cytolytic antitumor activity of CD4^+^ helper, CD8^+^ cytotoxic T, and natural killer (NK) cells (Figure 2). Furthermore, high extracellular ADO levels can impact on the antigen-presenting activity of dendritic cells (DCs) and can activate immunosuppressive cells such as Tregs, myeloid-derived suppressor cells (MDSCs), and M2 macrophages. Actions of extracellular ADO are mediated upon binding to surface receptors, mainly the A2A receptor on immune and cancer cells. HIF-1α-sensitive A2A receptors are coupled to G_s_-proteins, activate adenylylcyclase, and increase intracellular cyclic adenosine monophosphate (cAMP) levels. A more detailed description of the relevant suppressive effects of ADO on the antitumor immune responses has been presented in Ref. (15).

## Lactate Accumulation Impedes Antitumor Immunity

Lactate (lac^−^) accumulation (up to 40 mM in heterogeneously distributed subvolumes of human cancers) (5) is a secondary consequence of hypoxia upon HIF-1α-induced reprogramming of metabolic pathways [e.g., upregulation of the glycolytic enzyme lactate dehydrogenase A (LDH-A) and accelerated glycolysis, “Warburg effect”]. Lactate *per se*, plentiful in hypoxic TMEs, mainly blocks proliferation, tumor infiltration, and cytokine production of T cells, inhibits the cytotoxic activity of NK, NKT, and CD8^+^ T cells, and increases the number of MDSCs that inhibit NK cell mediated cytotoxicity. Lactate, thus, can strongly repress antitumor immunity (18–21). In general, lactate actions are initiated after binding to the cell surface lactate receptor, GPR81 (syn. HCA-1), G_i_-mediated signaling and subsequent decrease of cAMP levels.

## Acidosis Opposes Antitumor Immune Responses

Extracellular acidosis in tumors mainly is a consequence of (a) an upregulation/acceleration of glycolysis [“Warburg effect,” associated with intensive conversion of glucose to lactic acid and inefficient adenosine triphosphate (ATP) production, but importantly very fast energy supply], (b) intensified glutaminolysis, (c) ketogenesis, (d) increased ATP-hydrolysis, (e) hydration of CO_2_ derived from oxidative metabolism and highly active pentose phosphate pathway, and (f) bicarbonate depletion in the extracellular space. Major mechanisms involved in the immune-suppressive action of acidosis (extracellular pH ≤ 6.8) include an inhibition of the proliferative and cytotoxic activity of NK cells and CD8^+^ T cells, secretion of TH1-type cytokines (e.g., IFN-γ) and a reduction in the expression density of T cell receptors (TCRs) (21–27).

## VEGF Overexpression and VEGF-R Activation Counteract Antitumor Immunity

Vascular endothelial growth factor is one of the major players that drives tumor angiogenesis and, therefore, supports tumor progression and metastasis (28). Hypoxia-/HIF-1α directly or indirectly (in the latter case *via* ADO, lactate or acidosis) can drive the expression of VEGF and activate VEGF-R, thus promoting tumor evasion from immune surveillance (24–27). VEGF also can negatively affect growth and maturation of immature granulocyte–macrophage progenitors and DC precursors and, thus, prevent T cell stimulation. In addition VEGF can recruit immunosuppressive cells such as M2 macrophages into the tumor stroma which give raise to tumor-associated macrophages with immunosuppressive capacity (29). Other VEGF-triggered immunosuppressive mechanisms are mediated through Treg cells, pro-tumor M2 macrophages, MDSCs, and/or the presentation of immune checkpoint inhibitors, such as PDL1 or CTLA-4 on tumor and effector cells. Antiangiogenic therapy, using inhibitors targeting the VEGF/VEGF-R pathway may also exert beneficial effects on the reactivation of immune responses (in addition to the debatable “normalization of the tumor vasculature” theory), as discussed recently (15).

## Externalization of PS Stimulates Immune-Suppressive Mechanisms

Under non-stress conditions, PS is selectively found on the inner leaflet of plasma membranes. Following environmental stress or in aging cells PS gets externalized to the outer membrane leaflet by scramblases. Externalized PS on dying normal cells acts as a dominant, anti-inflammatory eat-me signal for phagocytosis (efferocytosis) that allows controlled apoptotic cell death (30). Pathologically, the immunosuppressive activity of externalized PS has been converted into pro-inflammatory signals that can also support tumor progression. Upon hypoxic stress, the localization of PS is severely dysregulated in tumor and stromal cells (infiltrating MDSCs within the tumor stroma included) (30). If the controlled apoptotic clearance of tumor cells fails, secondary necrosis can be initiated which in turn can induce chronic inflammation and autoimmunity (31). A rapid removal of PS exposing apoptotic by phagocytosis is pivotal to prevent inflammatory responses and the maintenance of tolerogenic signals during homeostasis which in turn can downregulate antitumor immune responses. Furthermore, binding of PS exposing tumor-derived exosomes to PS-receptors (e.g., TIM-receptors on immune cells) (32), can also induce evolutionary conserved immune-suppressive signals (enhanced TGF-β and interleukin-10 secretion) that can further inactivate antitumor immune responses.

## Immune Suppression by Elevated Extracellular K^+^

Tumor cell death (apoptosis or necrosis as a consequence of sustained hypoxia) leads to an egress of intracellular ions into the extracellular compartment impacting on antitumor T cells, on many other immune cells, and on the efficacy of immune therapies for cancer patients (33, 34). High K^+^ concentrations in the interstitial space [approx. 40 mM (physiological range: 3.5–5 mM)] can impair Akt–mTOR phosphorylation signaling of the TCR, thus inhibiting production of effector cytokines by T cells (e.g., IFN-γ). These recently published data have identified a novel “ionic checkpoint blockade” acting on T cell effector function upon release of cellular contents during tumor cell death (33, 34).

## Therapeutic Strategies Counteracting the Immunosuppressive Activities of Hypoxia-Associated Factors in the TME

Therapeutic strategies alleviating immunosuppressive (and pro-tumor) activities of *ADO* have been described earlier [(14, 15) with Supplementary Material http://journal.frontiersin.org/article/10.3389/fimmu.2016.00332]. In a most recent communication, Vijayan et al. have extensively reviewed the current approaches (35).

Targeting immunosuppressive actions of *VEGF* have been summarized in depth earlier (15, 28, 36, 37).

Therapeutic targets inhibiting immunosuppressive activities of *lactate* have recently been discussed (20, 38, 39). Promising strategies include inhibition and knockdown of monocarboxylate transporters, small molecule LDH-A inhibitors, and inhibition of the cell surface lactate receptor GPR81 (40, 41).

Treatments targeting *tumor acidosis* include small molecules and antibodies interfering with pH regulating systems. Major regulators involved are several H^+^ transporters, proton pump ATPases, and carbonic anhydrases CAIX and CAXII, which can be blocked using corresponding pharmacological inhibitors (42). Preventing acidosis based on systemic buffer therapy using bicarbonate, imidazoles, or lysine has improved responses to immune therapies (22, 43). The use of inhibitors of CAIX and other new promising approaches offering new possibilities have recently been discussed in detail (44, 45).

Reversal of the *PS*-induced antitumor immunosuppression can be stimulated by PS-targeting therapeutics [e.g., Anx A5, bavituximab, see Table S1B in Supplementary Material (15)].

Impaired T cell functions following *egress of intracellular K^+^* into the interstitial space upon tumor cell death can be reversed via the overexpression of voltage-gated K^+^ channels type K_v_1.3 (34).

## Conclusion

From the data presented, it is evident that hypoxia-/HIF-1α-driven features of the TME, such as ADO and lactate accumulation, extracellular acidosis, VEGF overexpression, and VEGF-R activation, and PS-externalization from the inner to the outer leaflet of tumor cells or tumor-derived exosomes are accomplices (“fatal sextet” of TME) sabotaging spontaneous and therapeutically induced antitumor immune responses. Therapeutic strategies counteracting the immune-suppressive activities of these adverse factors have been reviewed recently (14, 15).

## Author Contributions

PV and GM equally contributed to the writing of this minireview.

## Conflict of Interest Statement

The authors declare that the research was conducted in the absence of any commercial or financial relationships that could be construed as a potential conflict of interest.

## Abbreviations

ADO, adenosine; A2AR, A2BR adenosine receptors; Anx A5, annexin A5; ATP, adenosine triphosphate; cAMP, cyclic adenosine monophosphate; CD39, ectonucleoside triphosphate diphosphohydrolase 1; CD73, ecto 5′-nucleotidase; CD4^+^, helper T cell; CD8^+^, cytotoxic T cell; DC, dendritic cell; ENT-1, equilibrative nucleoside transporter 1; G_s_, G_i_ stimulatory/inhibitory G-protein; GPR81, G-protein receptor 81 (cell surface lactate receptor); HCA-1, hydroxycarboxylic acid receptor 1 (syn. GPR81); HIF, hypoxia-inducible (transcription) factor; IL, interleukin; IFN-γ, interferon γ; LDH-A, lactate dehydrogenase A; MCT, monocarboxylate transporter; Mϕ, macrophage; MDSC, myeloid-derived suppressor cell; mTOR, mechanistic (“mammalian”) target of rapamycin; NK, natural killer cell; NKT, natural killer-like T cell; PANX, pannexin (ATP channel); PDL1, programmed cell death 1 protein ligand; pO_2_, oxygen partial pressure; PS, phosphatidylserine; ROS, reactive oxygen species; TCR, T cell receptor; TGF-β, transforming growth factor β; TAM, tumor-associated macrophage; TIM, T cell immunoglobulin and mucin domain (PS surface receptor); TME, tumor microenvironment; TNF-α, tumor necrosis factor-α; Tr1, Type 1 regulatory T cell; VEGF, vascular endothelial growth factor; VEGF-R, VEGF receptor.
